# Curing Behaviors of Alkynyl-Terminated Copolyether with Glycidyl Azide Polymer in Energetic Plasticizers

**DOI:** 10.3390/polym12051199

**Published:** 2020-05-25

**Authors:** Jinghui Hu, Yina Liu, Kun Cong, Jiyu He, Rongjie Yang

**Affiliations:** School of Materials Science and Engineering, Beijing Institute of Technology, Beijing 100081, China; 3120170524@bit.edu.cn (J.H.); 7420180225@bit.edu.cn (Y.L.); ckkc_87@126.com (K.C.); hejiyu@bit.edu.cn (J.H.)

**Keywords:** polytriazole polyethylene oxide-tetrahydrofura (PTPET), LF-NMR, crosslinking, A3, Bu-NENA

## Abstract

Alkynyl-terminated polyethylene oxide−tetrahydrofuran (ATPET) and glycidyl azide polymer (GAP) could be linked through click-chemistry between the alkynyl and azide, and the product may serve a binder for solid propellants. The effects of the energetic plasticizers A3 [1:1 mixture of bis-(2,2-dinitropropy) acetal (BDNPA) and bis-(2,2-dinitropropyl) formal(BDNPN)] and Bu-NENA [N-butyl-N-(2nitroxyethyl) nitramine] on the curing reaction between ATPET and GAP have been studied. A diffusion-ordered nuclear magnetic resonance spectroscopy (DOSY-NMR) approach has been used to monitor the change in the diffusion coefficient of cross-linked polytriazole polyethylene oxide−tetrahydrofuran (PTPET). The change in the diffusion coefficient of PTPET with A3 plasticizer is significantly higher than that of PTPET with Bu-NENA. Viscosity analysis further highlighted the difference between A3 and Bu-NENA in the curing process—the curing curve of PTPET (A3) with time can be divided into two stages, with an inflection point being observed on the fourth day. For PTPET (Bu-NENA), in contrast, only one stage is seen. The above methods, together with gel permeation chromatography (GPC) analysis, revealed distinct effects of A3 and Bu-NENA on the curing process of PTPET. X-ray Photoelectron Spectroscopy (XPS) analysis showed that Bu-NENA has little effect on the valence oxidation of copper in the catalyst. Thermogravimetric (TG) analysis indicated that Bu-NENA helps to improve the thermal stability of the catalyst. After analysis of several possible factors by means of XPS, modeling with Material Studio and TG, the formation of molecular cages between Bu-NENA and copper is considered to be the reason for the above differences. In this article, GAP (*Mn* = 4000 g/mol) was used to replace GAP (*Mn* = 427 g/mol) to successfully synthesize the PTPET elastomer with Bu-NENA plasticizer. Mechanical data measured for the PTPET (Bu-NENA) sample included *ε* = 34.26 ± 2.98%, and *σ* = 0.198 ± 0.015 MPa.

## 1. Introduction

Nowadays, the most widely used types of propellants include double-based propellant, butyl-hydroxyl propellant, NEPE propellant [NitrateEster Plasticized Polyether (NEPE) Propellant], and so forth. A3 [a 1:1mixture of bis-(2,2-dinitropropy) acetal (BDNPA) and bis-(2,2-dinitropropyl) formal (BDNPF)] and Bu-NENA [N-butyl-N-(2nitroxyethyl) nitramine] are two commonly used plasticizers in composite solid propellants. [1,2,3] These two plasticizers are widely applied in double-based and gun propellants, significantly improving their energy characteristics. [2,4] Recently, higher requirements have been placed on the performance indicators of solid composite propellants. It is hoped that they can be applied to NEPE propellants in order to further increase their energetic potential.

Click chemistry is a kind of classical chemical reaction between the azide and alkynyl groups and has been widely exploited since its discovery by Sharpless in 2001. [5] Its advantages include no side reaction, rapidity, high efficiency and no reaction with water, and so on. [6,7] This reaction has already been successfully applied in many fields, for example, biomedicine, polymer synthesis, [8] solid phase reaction, [9] and so forth. [10,11] Polytriazole polyethylene oxide−tetrahydrofuran (PTPET) elastomer is an example of a propellant successfully assembled by click chemistry in. [12,13] This elastomer is prepared from alkyne-terminated polyethylene oxide tetrahydrofuran co-polyether (ATPET) and glycidyl azide polymer (GAP). It has the potential to be used in NEPE propellants. However, an issue arises when A3 and Bu-NENA plasticizers are applied in PTPET-based propellant. Compared to those with A3, elastomers and propellants including Bu-NENA cannot be successfully cured into finished products. This phenomenon indicates that the curing system of PTPET including Bu-NENA plasticizer [PTPET (Bu-NENA), same as below] still has some problems, which limit its application in propellants. No published data on this issue are yet available. It is important to understand the role of the Bu-NENA in the curing process of the PTPET elastomer.

Calorimetry analysis is a classical method that can be used to evaluate the curing behavior of thermosetting elastomers. [14,15] In this study, it was necessary to analyze the effects of two plasticizers on the curing performance. The amounts of plasticizer used in the experiments were large, and their physical properties were quite different. Hence, it was impossible to obtain reliable information on curing of the elastomer by calorimetric analysis. However, Diffusion-ordered nuclear magnetic resonance spectroscopy (DOSY-NMR) has become a powerful tool for studying molecular interactions in solutions and determining molecular size. [16,17,18] It has also received increasing attention from chemical researchers, especially for exploring the interactions between subject and object, [19,20] molecular aggregation and other fields. [21] Diffusion is an inherent property of all molecules and varies with the size, shape, solubility, charge distribution, and so forth, of the particles. The nature of a mixture can also be analyzed by DOSY-NMR based on their diffusion coefficient of the proton environment.

In this article, we aim to confirm the effect of Bu-NENA on the curing of terminal alkynyl polytetrahydrofuran ethylene oxide copolyether and GAP by means of DOSY-NMR, viscosity and GPC analytical methods. Meanwhile, we discuss the potential effect of the presence of the Bu-NENA in PTPET. It is hoped that the collected data may be helpful in solving the above mentioned problems by comparative analysis of experimental data and the results of theoretical calculations for the systems with A3 and Bu-NENA.

## 2. Experimental

### 2.1. Materials and Methods

**Scheme 1:**
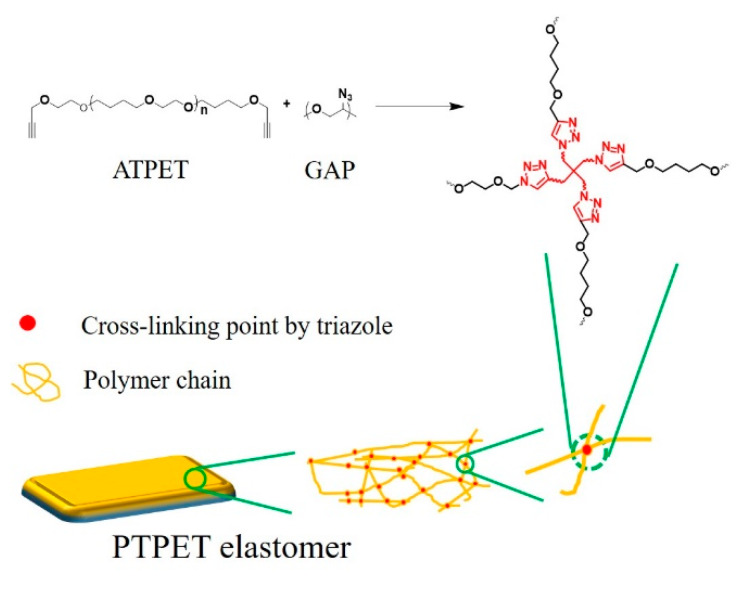
Synthesis of polytriazole polyethylene oxide−tetrahydrofuran (PTPET) elastomer.

ATPET (*M_n_* = 4000 g/mol) was synthesized in our laboratory. [22] GAP (*M_n_* = 427 g/mol, the average functionality of −N_3_ was 3.8) and GAP (*M_n_* = 4000 g/mol, the average functionality of −N_3_ was 38) was used as the curing agent and offered by Liming Chemical Research Institute. A3 and Bu-NENA were the two plasticizers used in this experiment provided by Xi’an North Huian Chemical Industry Co., Ltd. (Xi’an, China). The mass ratio of plasticizer to ATPET in this experiment is 1:1. A mole ratio of 1:1 for the azide groups to the alkynyl groups was obtained by using GAP (*M_n_* = 427 g/mol), [23] whereas 10:1 was achieved by using GAP (*M_n_* = 4000 g/mol) experiment. The PTPET elastomer was synthesized by the click-chemistry reaction between ATPET and GAP (*M_n_* = 427 g/mol) in the presence of a copper catalyst at 50 °C for 7 days. A schematic diagram of the synthesis method for the PTPET elastomer is shown in Scheme 1 [13].

### 2.2. Measurements and Analysis

Nuclear magnetic resonance (NMR) spectra were acquired on a Bruker Avance HD 600 instrument (Bruker, Karlsruhe, Germany) at 299.15 K. The ledbpgp2s1d spin echo sequence was applied for DOYS-NMR experiments. Sixteen points were recorded to produce each ^1^H-NMR spectrum. The delay between experiments (d1) was set to 1 s. (D16: 0.0002 s, D20: 0.5 s, D21: 0.005 s, GPZ7: −17.13%, GPZ8: −13.17%, P100: 600, P30: 1000). Each prepared sample (100 mg) was added to a 5 mm NMR tubes containing 0.6 mL of CDCl_3_. The viscosity of each reaction system was measured with a HAAKE MARS iQ apparatus (Thermo Fisher Scientific, Waltham, MA, USA). Molecular weights and their distributions were determined by gel permeation chromatography (GPC, Waters-1515, Atlanta, GA, USA). Thermogravimetric analysis (TGA) was performed on a Netzsch 209 F1 instrument (Selb, Germany, Al_2_O_3_ crucible) instrument with the following test conditions—test atmosphere N_2_, scanning rate 10 °C/min and temperature range 40~500 °C. X-ray photoelectron spectroscopy (XPS) was performed on a PHI QUANTERA-II SXM (ULVAC-PHI, Kanagawa, Japan) apparatus using the C1s peak (B.E. = 284.6 eV) as a reference.

**Figure 1:**
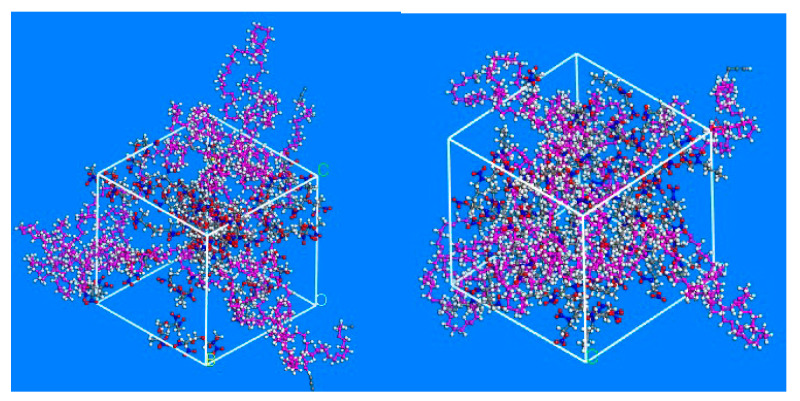
Chemical models of Alkynyl-terminated polyethylene oxide−tetrahydrofuran (ATPET) & A3 (bis-(2,2-dinitropropy) acetal (BDNPA)/bis-(2,2-dinitropropyl) formal (BDNPF) = 1/1) (**left**) and ATPET & Bu-NENA (**right**).

Molecular dynamics (MD) simulation analysis of the compatibility and interaction between the plasticizers A3/Bu-NENA and polymer was performed using the amorphous builder and forcite modules in Material Studio (MS) 6.0 software. Molecular models of ATPET binder with Bu-NENA, and A3 (composed of BDNPF and BDNPA mixed in equal mass ratio) were established according to their respective structural formulae (as shown in Figure 1) by MS. The mass ratio of ATPET to plasticizer in the model is 1:1. The detailed experimental parameters of MS simulation were based on using the reference parameters. [24] (Forcefield: Compass II, Buffer width: 0.5 Å, Cutoff distance: 12.5 Å).

## 3. Results and Discussion

### 3.1. H-NMR and DOSY Analysis

**Figure 2:**
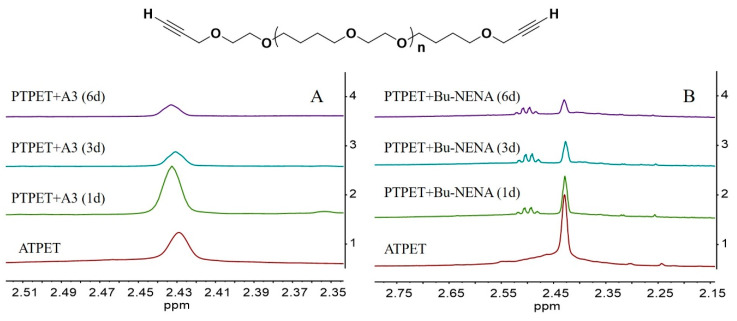
Temporal evolution of the ^1^H-NMR spectra of the PTPET+ A3 (**A**) and PTPET+ Bu-NENA (**B**) systems.

Extensive peak overlap precluded the identification of single components in the mixtures by ^1^H-NMR. However, some information could still be gleaned from non-overlapping peaks. The peak at 2.45 ppm can be ascribed to the signal of the hydrogen atom on the terminal alkynyl group in ATPET. The changes in the ^1^H-NMR spectra with time for the reaction systems with plasticizers A3 and Bu-NENA are shown in Figure 2. It is clear that for both plasticizer systems, the intensity of this peak decreases with time. This phenomenon is consistent with partial consumption of the terminal alkynyl groups through click reaction with the azide groups in ATPET.

Clearly, it was difficult to unequivocally identify and quantify the individual components in this complex system by ^1^H-NMR. [25] To get more information, high-field nuclear magnetic resonance (NMR) diffusion equipment was used to monitor the phase transition molecular dynamics during polymer formation occurring in the liquid systems containing the respective plasticizer. DOSY-NMR is an effective approach for separating overlapping peaks by plotting chemical shift against the diffusion coefficient. In previous literature, an empirical formula between diffusion coefficient and molecular weight has been established, allowing different substances to be distinguished. [26] The DOSY-NMR method has proven to be very valuable for identifying components in unknown samples.

**Figure 3:**
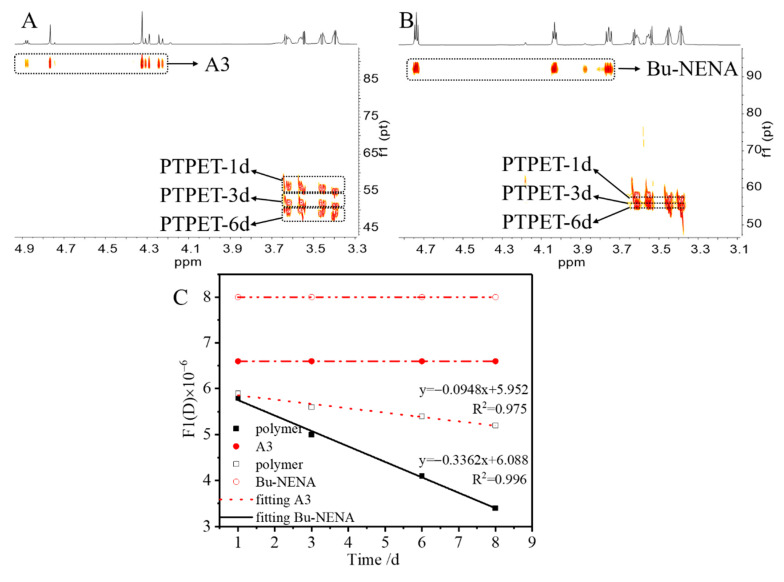
DOSY-NMR spectral analysis of PTPET/plasticizer mixtures at different times. (**A**) A3 system, (**B**) Bu-NENA system, (**C**) Relationships between diffusion coefficient and time for the above two reaction systems.

DOSY spectra of the plasticizers and polymer mixtures after different times are shown in Figure 3; (A) A3 and polymer; (B) Bu-NENA and polymer. All samples were tested at the same concentration, with the same test parameters and NMR pulse sequence. The experimental results of different test time were superimposed on an analysis. The DOSY spectra indicated good separation of the plasticizer and polymer in the reaction solution. The peaks in the dashed boxes showed very similar diffusion coefficients, implying that they belonged to the same compound. The signals in the upper dashed boxes in Figure 3A,B are those of the plasticizers A3 and Bu-NENA, respectively, and their diffusion coefficients do not change with time. They have larger diffusion coefficients than that of the polymer. The signals in the bottom right-hand corners of Figure 3A,B are those of the PTPET polymer. By comparing and analyzing these results, it can clearly be seen that the degrees of polymerization in the presence of the respective plasticizers are significantly different, in accordance with the correlation between the molecular weight of a polymer and its diffusion coefficient. [27] The diffusion coefficient of the polymer in the A3 system is significantly larger than that in the Bu-NENA, indicating that the polymer produced in the former system is much larger. In order to further understand the differences between the respective systems, the relationship between diffusion coefficient and time or each substance was analyzed, and the results are shown in Figure 3C. The relationship between the diffusion coefficient of the polymer and time in the A3 system can be expressed as y = −0.0948x + 5.952, with *R*^2^ = 0.975, whereas that in the Bu-NENA system can be expressed as y = −0.3362x + 6.088, with *R*^2^ = 0.996.

### 3.2. Viscosity Analysis

**Figure 4:**
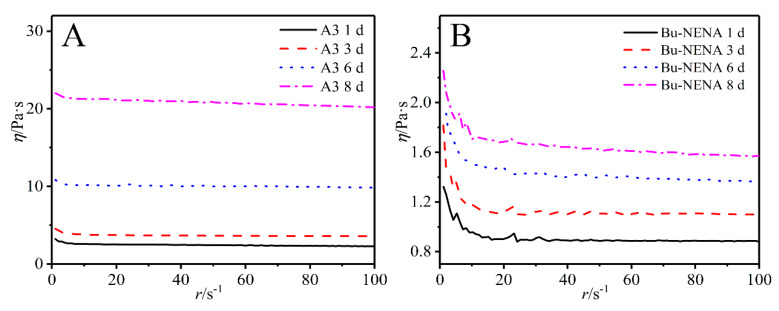
Viscosity curves of PTPET with A3 (**A**) and Bu-NENA (**B**) after different times.

The intrinsic viscosities of samples of the A3 and Bu-NENA systems were determined by stress rheometer measurements in the range 1–100 rad/s. The main linkage of the s molecular chain of the ATPET is the C-O bond. Consequently, the internal rotation barrier is small, the molecular chain is flexible, and the fluidity of the material is also good. Viscosity change curves with respect to time are shown in Figure 4, for the A3 (A) and Bu-NENEA (B) systems. With increasing reaction time, the molecular weight of the PTPET polymer gradually increases due to the click chemistry reaction between ATPET and GAP. It is well known that the viscosity of a polymer is a function of molecular weight, shear conditions, temperature, and pressure [28]. Curves A and B in Figure 4 verify that the viscosities of both polymer systems gradually increased with reaction time. However, distinct trends can be discerned for the respective systems. The viscosity increase of the A3 system was significantly greater than that of the Bu-NENA system.

In order to further analyze the change trends of the viscosity in the two systems, the data were subjected to statistical analysis, and depicted in Figure 4. The collected data points were utilized to fit a plot of log_2_*η* vs. time. Results for the Bu-NENA system show a good linear regression; the fitting function is y = 0.1120x − 0.2424, with *R* = 0.993. However, there are obvious differences for the A3 system. The first half of the data shows as a good fitting linear relationship, for which the fitting function is y = 0.3116x + 0.8887, with *R* = 0.999. The second half of the data shows another good linear fitting relationship, the fitting function for which is y = 0.5656x − 0.0956, with *R* = 0.989.

According to the theory of the polymer material flow, molecular weight is one of the main factors affecting the rheological properties of polymers. [29] The relationship between the molecular weight and viscosity is expressed by the simple equations:

(1) $$\eta_{0}=K_{1}M_{W}\;(M_{W}<M_{c})$$

(2) $$\eta_{0}=K_{2}M_{W}^{3.4}\;\left(M_{W}>M_{c}\right),$$

where *Mc* is the critical molecular weight of the polymer, which is related to the molecular weight restricted by the entanglement of polymer segments.

**Figure 5:**
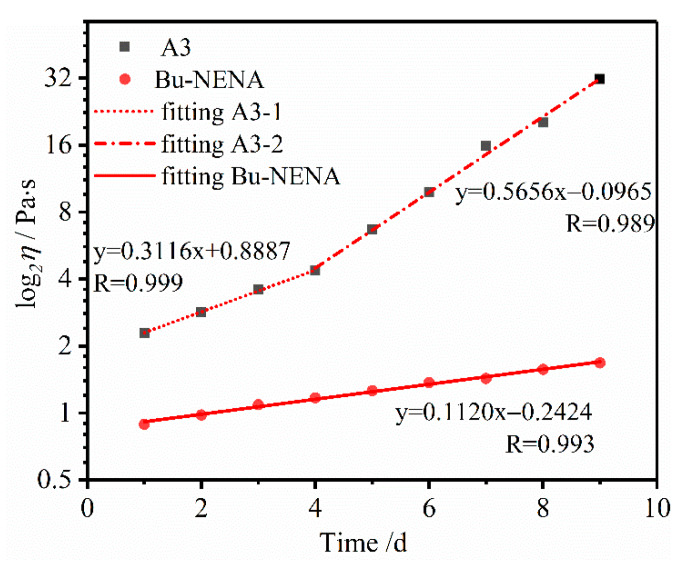
Viscosity data and fitting lines for PTPET with Bu-NENA and A3 at various time.

Once the molecular weight exceeds *Mc*, mutual entanglement occurs between the molecular chains. Interactions between the molecular chains suddenly increase due to this entanglement, and the stress on one molecular chain will be transferred to other molecular chains, significantly increasing the viscosity of the polymer. As shown in Figure 5, an inflection point in the viscosity curve of the A3 system is seen after 4 days. This experimental phenomenon implies that the molecular weight of the polymer in A3 could exceed the gel point. This is an important factor with regard to the application of A3 and Bu-NENA plasticizers in PTPET-based propellant. A3 elastomer and propellants can be successfully cured into final products, but systems with Bu-NENA cannot.

### 3.3. Gel Permeation Chromatography (GPC) Analysis

**Figure 6:**
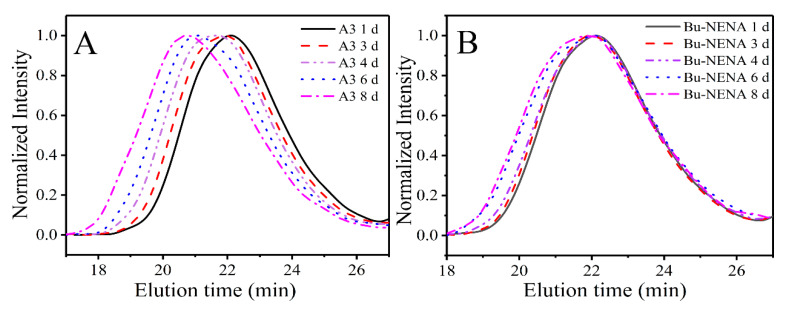
Gel permeation chromatography (GPC) curves of the uncrosslinked polymer in PTPET after different times, (**A**) A3 and (**B**) Bu-NENA.

Gel permeation chromatography (GPC) is an effective technique for characterizing the molecular weight changes of polymers. In order to further corroborate the validity of the viscosity analysis, the molecular weight changes of the polymer in the A3- and Bu-NENA-containing systems were tracked, and the results are presented in Figure 6. 10 mg of the polymer sample and 0.5 mL of THF were added to a centrifuge tube. After the sample is fully dissolved, the mixture was filtered with a 0.2 μm filter membranes and take the filtrate for analysis and testing. Thermosetting elastomers can be formed by linear polymers through cross-linking reactions. Therefore, the molecular weight of the uncrosslinked portion will gradually increase with the increase of reaction time and reaction degree, and the polydispersity index (PDI) will gradually become wider. The GPC analysis results for the two systems show that the peak time of the polymer shifts to the left with increasing reaction time. From Figure 6A,B, it can be seen that there was a significant difference in the molecular weights of the polymers in the respective systems. The amplitude of the left shift of the curve for the A3 system is significantly higher than that for the Bu-NENA system, and the polydispersity index (PDI) is also wider for the former than for the latter. The *Mn* and PDI analysis data of the studied samples are shown in Table 1. Although the GPC data obtained for the polymers related to the uncrosslinked part of the system, the results are still somewhat enlightening. GPC analysis data are consistent with the viscosity and DOSY NMR analysis described above.

Through the above experimental analysis by NMR, GPC and viscosity, it is confirmed that there are indeed differences between the A3- and Bu-NENA-containing systems. In order to further delineate the reasons for the differences between the above two systems, the following sections outine several possibilities and gives the corresponding analysis and characterization data.

### 3.4. X-ray Photoelectron Spectroscopy Analysis

**Figure 7:**
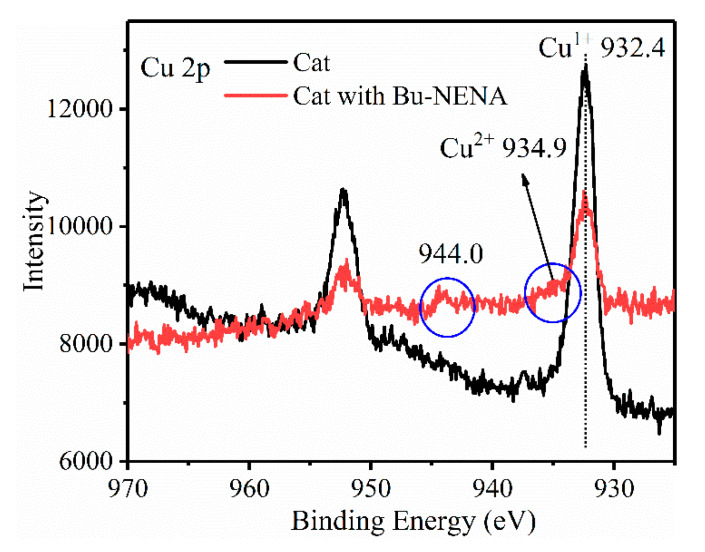
X-ray photoelectron spectroscopy (XPS) analysis of pure catalyst and Bu-NENA soaked catalyst.

One possibility is that Bu-NENA inactivates the catalyst and oxidizes monovalent copper to divalent copper, which may cause failure of the click chemical reaction. In order to test this hypothesis, a catalyst sample, 10 mg was immersed in Bu-NENA (1 mL). The mixture was placed in an oven at 50 °C for 5 d, then vacuum-dried and sampled for XPS analysis. XPS results for the pure catalyst and the Bu-NENA soaked catalyst are displayed in Figure 7. Only two peaks were seen in the XPS analysis of the pure catalyst, namely the 2p 1/2 peak of Cu^+^ in the catalyst at 952.3 eV and the 2p 3/2 peak at 932.4 eV. [30] The catalyst treated with Bu-NENA showed a small peak at 934.9 eV attributable to Cu^2+^, and a small satellite peak at 944.0 eV. However, the signal intensities of these two peaks were only slightly higher than the signal-to-noise ratio. This observation indicated that only a small fraction of the Cu^+^ in the catalyst soaked with Bu-NENA was oxidized to Cu^2+^. It could be observed from the peak areas that most of the Cu^+^ had not been oxidized and was still monovalent. The samples treated with Bu-NENA and the original sample both showed the green color of monovalent copper. The above analysis shows that oxidation of the catalyst from Cu^+^ to Cu^2+^ by Bu-NENA was not the main reason for suppression of the reaction.

### 3.5. Solubility Parameter Analysis

Miscibility refers to the property of a polymer and plasticizer to form a macroscopically homogeneous material. The parameter of cohesive energy density (*CED*) has a direct effect on the physical characteristics of the polymer. The solubility parameter (*δ*) plays an important role in the theory of mixtures. [31] There is also speculation that the compatibility of ATPET with Bu-NENA and A3 plasticizers is different, making the reactant system unable to mix uniformly, resulting in the observed reactivity difference.

The *CED* and *δ* parameters of the two systems were calculated using molecular dynamics, and the results are shown in Table 2. The *CED* values of Bu-NENA and A3 are both over 420 J cm^−3^, classifying them as polar plasticizers. The ATPET matrix, meanwhile, is a low-polarity polymer, since its *CED* is 338.9 J cm^−3^. In our study, it is clear that the magnitudes of Δδ between the two polymers and plasticizer systems are < 2.1 (J cm^−3^)^0.5^. Based on the miscibility principle of similar material structures, when *δ* of the additive is close to that of the polymer, they can dissolve well in one another. Therefore, the probability of this explanation is low.

*CED* = *CED_v_* + *CED_e_* (3)

*δ* = *δ_v_* + *δ_e_*. (4)

However, this approach is not suitable for systems with strong hydrogen bonds between the components. This is because the theory of solubility parameters only considers the influence of intermolecular dispersion forces, and ignores dipole forces and hydrogen-bonding interactions.

### 3.6. Radial Distribution Function Analysis

Intermolecular interactions are strongly influenced by steric effects. The radial distribution function (RDF) can be used to reveal the interactions of different atoms of ATPET with plasticizers.

The distance at which a peak appears an effective approach for inferring the type of interaction force. Generally, the van der Waals force is from 0.31 nm to 0.50 nm, while the distances of hydrogen bonds are from 0.26 nm to 0.31 nm. [32,33] The value of the force is judged by the peak height in the RDF curve.

**Figure 8:**
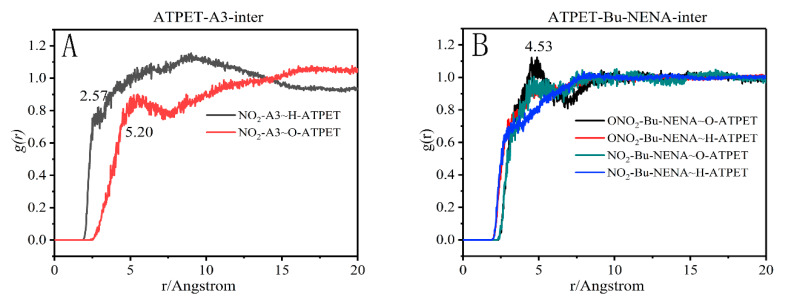
Radial distribution functions (RDFs) for different atoms of PTPET & A3 (**A**) / PTPET & Bu-NENA (**B**).

The RDF curves for interactions between specific moieties of ATPET and the plasticizers (A3/Bu-NENA) are shown in Figure 8. There is a peak at 2.57 Å which *g(r)* = 0.81 for the pair of H in ATPET and the NO_2_ group in A3; the peak for O in ATPET and the NO_2_ group in A3 is at 5.20 Å which *g(r)* = 0.89. In the RDF curves for the Bu-NENA system, there is a peak at 4.53 Å which *g(r)* = 1.00 for the pair of O in PTPET and the NO_2_ group in Bu-NENA; the peak for H in ATPET and the NO_2_ group in Bu-NENA is at 2.91 Å which *g(r)* = 0.69. Meanwhile, for the relationship between the ONO_2_ group and atoms in ATPET, there is a main peak at 4.53 Å which *g(r)* = 1.12 for the pair of O in ATPET and the ONO_2_ group in Bu-NENA; the peak for H in ATPET and the ONO_2_ group in Bu-NENA is at 3.35 Å which *g(r)* = 0.79. From comprehensive analysis of the RDF curves, the interaction forces of NO_2_-A3~H-ATPET and NO_2_-Bu-NENA~H-ATPET are hydrogen bonds, while the interaction forces of NO_2_-A3~O-ATPET, NO_2_-Bu-NENA~O-ATPET, ONO_2_-Bu-NENA~O-ATPET and ONO_2_-Bu-NENA~H-ATPET are van der Waals interactions. The highest peak probably for a strong interaction force is between the ONO_2_ group of Bu-NENA and the O in ATTPE. The value of *g(r)* for the Bu-NENA system sharply increased by about 2.5 Å, indicating that the effect of hydrogen-bonding was greater than in the A3 system.

### 3.7. Thermogravimetric (TG) Analysis

According to the above experimental and computational analyses, it is clear that the above three possibilities are not the main factors that prevent PTPET from curing. From previous literature on the CuAAC reaction, a specific phenomenon is that copper complexes are more susceptible to inhibition by forming molecular cages in some ligand environments. [34] Bu-NENA competes with the complexing ligand in the copper catalyst, inactivating the copper center and preventing the CuAAC reaction.

**Figure 9:**
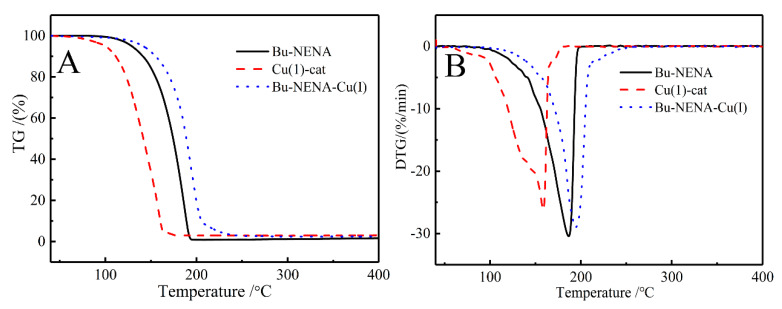
TG (**A**) and derivative thermogravimetric analysis (DTG) (**B**) curves of Bu-NENA and catalyst.

As the ONO_2_ group in Bu-NENA is electron-donating, it is speculated that Bu-NENA inhibits catalysis of the CuAAC reaction through the formation of an inhibitory molecular cage with copper. The catalyst (10 mg) was dissolved in Bu-NENA (1mL) to form a homogeneous solution. A sample was then subjected to thermogravimetric analysis, and the results are exhibited in Figure 9. Thermal decomposition onset temperatures (*T*_95%_) for the respective samples were as follows—*T_cat-_*_95%_ = 99.9 °C, *T_Bu-NENA-_*_95%_ = 128.9 °C, *T_mix-_*_95%_ = 141.2 °C. The maximum decomposition temperatures (*T_m_*) were as follows—*T_cat-m_* = 158.6 °C, *T_Bu-NENA-m_* = 186.5 °C, *T_mix-m_* = 194.5 °C. The values of *T*_95%_ and *T_m_* for the sample were greatly improved after mixing. The above experimental data give supplementary evidence that Bu-NENA forms a complex with copper in the catalyst and modifies its properties.

### 3.8. An Alternative Method

How to apply Bu-NENA in solid propellants is still an issue that urgently needs to be addressed because the energy of Bu-NENA is higher than that of A3. In view of the fact that the above-mentioned molecular cage structure cannot perform CuAAC reaction, thermal catalysis of the reaction between an azide and an alkynyl group to form a triazole ring has become a possible solution.

**Figure 10:**
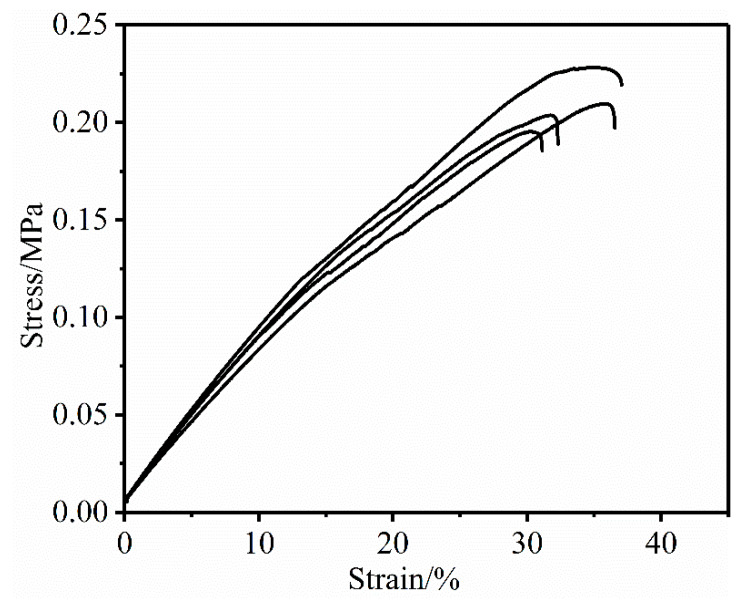
Stress-strain curves of PTPET (including Bu-NENA) with GAP (*Mn* = 4000 g/mol).

GAP (*Mn* = 4000 g/mol, *R* = 38) was used to replace GAP (*Mn* = 427 g/mol, *R* = 3.8) to form PTPET elastomer (including Bu-NENA) without catalyst. An PTPET (m_ATPET_: m_Bu-NENA_ = 1:1) elastomer was successfully formed after 7 days at 50 °C, and its mechanical properties was shown in Figure 10: *ε* = 34.26 ± 2.98%, *σ* = 0.198 ± 0.015 MPa. However, there is still a big gap for the mechanical properties between this container Bu-NENA elastomer and the PTPET elastomer without Bu-NENA. For the PTPET elastomer—*ε* = 228.45%, *σ* = 0.69 MPa. [35] The above data is also much lower than the A3 plasticized Glycidyl Azide Polymer-Polycaprolactone-Polyurethane elastomer system, which has a tensile strength of 1.8 MPa and a tensile strain at break of 1780%. [36] This experiment showed by using GAP (*Mn* = 4000 g/mol), the PTPET elastomer could be cured in the presence of plasticizer Bu-NENA. Although the mechanical properties of PTPET (including Bu-NENA) elastomers are still relatively low, this still has great significance. This is an example report of the successful preparation of PTPET (including Bu-NENA) elastomers.

## 4. Conclusions

DOSY-NMR, viscosity and GPC results have confirmed that there is indeed a considerable difference between the plasticizers A3 and Bu-NENA during the curing of PTPET. The curing reaction between ATPET and GAP is less efficient in the presence of Bu-NENA, but the precise mechanism needs further research. Monovalent copper in the catalyst is oxidized to a divalent state. Bu-NENA is decomposed to generate NO_2_, accounting for some differences between the properities of Bu-NENA and A3. Several possibilities for the differences between the two plasticizers A3 and Bu-NENA have been proven not to be the main factors that prevent PTPET from curing. The formation of molecular cages between the catalyst and Bu-NENA seems to be the most probable reason for the difference, but the specific mechanism needs further in-depth study. Finally, we propose a method of applying thermal catalysis to increase reaction sites after comprehensive analysis of the curing of azide and alkynyl groups. After a replacement of GAP (*Mn* = 4000 g/mol) with GAP (*Mn* = 427 g/mol), the curing of PTPET elastomer containing Bu-NENA plasticizer was successfully achieved.

It is hoped that this article gives an important indicator for the application of PTPET elastomers in composite propellants. However, the mechanical properties of the prepared elastomer are still relatively poor and cannot meet current production needs. Improving of the mechanical properties will be a further focus of our future work.

## Figures and Tables

**Table 1:** Analysis data of the uncrosslinked polymer samples in PTPET.

A3 Sample	*Mn* (g/mol)	*Mw* (g/mol)	PDI	Bu-NENA Sample	*Mn* (g/mol)	*Mw* (g/mol)	PDI
1 d	5341	10,159	1.66	1 d	5277	9957	1.68
3 d	5498	10,379	1.68	3 d	5343	10,748	1.69
4 d	5915	11,535	1.77	4 d	5376	10,793	1.72
6 d	6236	12,128	1.95	6 d	5897	11,494	1.81
8 d	6343	12,191	2.38	8 d	5947	11,546	1.83

**Table 2:** Cohesive energy density (*CED*) and *δ* of PTPET with Bu-NENA and A3.

Material	m (Bu-NENA:ATPET) = 1:1	m (A3/ATPET) = 1:1	ATPET
*CED/*(J cm^−3^)	426.2	423.9	338.9
*CED_v_/*(J cm^−3^)	335.3	297.6	296.3
*CED_e_/*(J cm^−3^)	74.66	110.5	263.5
*δ/*(J cm^−3^)^0.5^	20.65	20.59	18.40
*δ_v_/*(J cm^−3^)^0.5^	18.31	17.25	17.21
*δ_e_/*(J cm^−3^)^0.5^	8.64	10.51	5.13
